# MicroRNA-148a/b-3p regulates angiogenesis by targeting neuropilin-1 in endothelial cells

**DOI:** 10.1038/s12276-019-0344-x

**Published:** 2019-11-13

**Authors:** Hyejeong Kim, Yeongrim Ko, Hyojin Park, Haiying Zhang, Yoonjeong Jeong, Yeomyeong Kim, Minyoung Noh, Songyi Park, Young-Myeong Kim, Young-Guen Kwon

**Affiliations:** 1 Department of Biochemistry, College of Life Science and Biotechnology, Yonsei University, Seoul, 03722 Korea; 2 Vascular System Research Center, Kangwon National University, Chuncheon, Kangwon 24341 Republic of Korea

**Keywords:** Cell migration, RNAi

## Abstract

MicroRNAs (miRs) are crucial regulators of vascular endothelial cell (EC) functions, including migration, proliferation, and survival. However, the role of most miRs in ECs remains unknown. Using RNA sequencing analysis, we found that miR-148a/b-3p expression was significantly downregulated during the differentiation of umbilical cord blood mononuclear cells into outgrowing ECs and that decreased miR-148a/b-3p levels were closely related to EC behavior. Overexpression of miR-148a/b-3p in ECs significantly reduced migration, filamentous actin remodeling, and angiogenic sprouting. Intriguingly, the effects of decreased miR-148a/b-3p levels were augmented by treatment with vascular endothelial growth factor (VEGF). Importantly, we found that miR-148a/b-3p directly regulated neuropilin-1 (NRP1) expression by binding to its 3′-untranslated region. In addition, because NRP1 is the coreceptor for VEGF receptor 2 (VEGFR2), overexpression of miR-148a/b-3p inhibited VEGF-induced activation of VEGFR2 and inhibited its downstream pathways, as indicated by changes to phosphorylated focal adhesion kinase (FAK), extracellular signal-regulated kinase (ERK), and p38 mitogen-activated protein kinase. Collectively, our results demonstrate that miR-148a/b-3p is a direct transcriptional regulator of NRP1 that mediates antiangiogenic pathways. These data suggest that miR-148a/b-3p is a therapeutic candidate for overcoming EC dysfunction and angiogenic disorders, including ischemia, retinopathy, and tumor vascularization.

## Vascular biology: MicroRNA implicated in blood vessel formation

A small regulatory RNA molecule helps prevent the development of new blood vessels, a finding that could have implications for the treatment of vascular disease and cancer. Young-Guen Kwon from Yonsei University in Seoul, South Korea, and colleagues cataloged all the microRNAs expressed during the differentiation of umbilical cord blood stem cells into precursors of the cells that line the inside of blood vessels. One microRNA in particular stood out for its association with cellular differentiation. The authors showed that this microRNA, called miR-148a/b-3p, directly binds part of the gene transcript encoding neuropilin-1, thereby blocking the production of this receptor for growth factors involved in new blood vessel formation. Modulating the activity of miR-148a/b-3p could have therapeutic value for disorders marked by aberrant blood vessel growth.

## Introduction

Angiogenesis is the process by which new blood vessels form from the existing vasculature in response to angiogenic stimuli. Angiogenesis is crucial for ensuring healthy physiological processes, including embryo development and tissue repair, but it also has pathological roles in promoting tumor growth^1^. Vascular endothelial growth factor (VEGF), a very specific mitogen for vascular endothelial cells (ECs), stimulates angiogenesis by binding to VEGF receptor 1 (VEGFR1) and VEGF receptor 2 (VEGFR2) and their coreceptors, neuropilin-1 (NRP1) and neuropilin-2 (NRP2)^2^. In particular, VEGF/VEGFR2-induced signal transduction is a major ligand–receptor complex in the VEGF system. Activated VEGF/VEGFR2 signaling in ECs promotes proliferation, migration, cytoskeletal reorganization, differentiation, and the formation of new vasculature. Mounting evidence suggests that NRP1, which is a known coreceptor of VEGF, also plays an important role in angiogenesis. NRP1 can enhance VEGF-mediated activity and downstream signaling of VEGFR2 in ECs. Furthermore, the PDZ-binding domain in the cytoplasmic region of NRP1 is essential for both the formation of a stable NRP1/VEGFR2 complex^3,4^ and the trafficking of endocytosed VEGFR2^5^. Overexpression of NRP1 in tumor cells has also been shown to enhance tumor angiogenesis^6,7^.

MicroRNAs (miRs) are single-stranded, noncoding RNA molecules that have crucial roles in regulating gene expression; therefore, miRs control diverse cellular and metabolic pathways^8^. Recently, many studies have focused on identifying the relationship between a specific miR and the biological function of its target mRNA. Dysregulation of miRs leads to pathological processes, and some miRs are closely related to the onset of cancer because they modulate the expression of tumor suppressors. For example, miR-148a-3p is a tumor suppressor that is significantly downregulated in several types of cancers, including hepatocellular carcinoma, gastric cancer, and ovarian cancer^9–11^. miR-148b-3p is also reported to participate in the regulation of tumorigenesis. Indeed, miR-148b-3p represses tumor growth by regulating the proto-oncogene receptor tyrosine kinase, KIT, in gastrointestinal stromal tumors^12^. Although there has been extensive research on miR-148a/b in the context of cancer, its angiogenic properties remain largely unknown.

In the present study, we report the expression of miR-148a/b-3p during the differentiation of umbilical cord blood mononuclear cells (UCB-MNCs) into outgrowing ECs (OECs) and investigate the effects of miR-148a/b-3p on EC migration, F-actin remodeling, and angiogenic sprouting. We also identified target genes regulated by miR-148a/b-3p. Finally, we evaluated the role of miR-148a/b-3p in the VEGF-induced VEGFR2 signaling pathway. Our findings indicate that miR-148a/b-3p may be an interesting therapeutic target, particularly in vascular disease that is characterized by upregulated or aberrant angiogenesis.

## Materials and methods

### Isolation and culture of UCB-MNCs, OECs, and human umbilical vein ECs (HUVECs)

Approximately, 50 mL of human umbilical cord blood (HUCB) was collected by gravitational flow from umbilical cords that were still attached to the placenta. To separate UCB-MNCs from HUCB, the Ficoll–Paque density gradient method was used^13,14^. The isolated UCB-MNCs were resuspended in endothelial basal medium-2 (EBM-2, Clonetics Cell Systems). The complete media contained 20% heat-inactivated fetal bovine serum (FBS, HyClone), human VEGF, human fibroblast growth factor-B, human epidermal growth factor (EGFR), insulin-like growth factor 1 (IGF1), and ascorbic acid. Cells were seeded on human fibronectin (Sigma-Aldrich)-coated 6-well plates at a cell density of 2 × 10^7^ cells/well. After 3 days, nonadherent cells were washed away, and the media was changed every 2 days thereafter. After OEC colonies were established, samples were prepared for RNA-seq.

HUVECs were purchased from Lonza (C2517A; Walkersville, MD) and cultured in EGM-2 media (CC-3156, Lonza). The media was supplemented with the EGM-2 SingleQuot kit (CC-4176, Lonza), with 5% FBS, and 1% penicillin/streptomycin. Cells were routinely passaged at 80–90% confluency, and cells between passages 3 and 7 were used for experiments. Cells were maintained at 37 °C in a humidified atmosphere containing 5% CO_2_.

### HUVEC transfection with mimic-microRNAs

HUVECs (70% confluent) were transfected with miRIDIAN human hsa-miR-148a-3p or miRIDIAN human hsa-miR-148b-3p (Dharmacon) by incubating with Lipofectamine (Invitrogen) for 2 h and 30 min. As a control, the miRIDIAN microRNA Mimic Negative Control #1 was transfected into HUVECs at a concentration of 80 µM. Cells were transfected 48 h prior to the assay. The MiRIDIAN human hsa-miR-148a-3p-mimic microRNA sequence was: 5′-UCAGUGCACUACAGAACUUUGU-3′ and the MiRIDIAN human hsa-miR-148b-3p-mimic microRNA sequence was 5′-UCAGUGCAUCACAGAACUUUGU-3′. The MiRIDIAN microRNA Mimic Negative Control #1 sequence was 5′-UCACAACCUCCUAGAAAGAGUAGA-3′.

### MicroRNA (miR) identification by RNA sequencing analysis

Total RNA integrity was tested using an Agilent Technologies 2100 Bioanalyzer, and only RNA with an integrity number (RIN) value greater than 8 was used in further experiments. Small RNA (sRNA) sequencing libraries were prepared according to the manufacturer’s instructions (Illumina Small RNA Prep kit). Samples were sequenced with an HISEQ 2000 sequencing system (Illumina). The RNA sequencing data were used to select miRs that were expressed in both humans and mice (106 miRs). We then selected miRs that had not been previously studied in ECs (35 miRs) for further evaluation.

### Target prediction with bioinformatics

Computational predictions of miR-148a-3p and miR-148b-3p target genes were performed using the following published algorithms: DIANA-microT-CDS (http://www.microrna.gr/microT-CDS), TargetScan (http://www.targetscan.org), and miRDB (http://www.mirdb.org).

### RNA isolation, reverse transcription polymerase chain reaction (RT-PCR), and semiquantitative RT-PCR

Total miR was isolated using a miRNeasy Mini Kit (QIAGEN). RNA was isolated using Trizol (iNtRON), and RT-PCR of miRs was performed using 2× Maxima SYBR Green/ROX qPCR Master Mix (Thermo Scientific, K0221). All results were normalized to GAPDH expression levels. The PCR primers are listed in Table 1.

**Table 1:** Sequence of RT-PCR primers

hsa-miR-148a	3p	Stem–loop	5′-GTCGTATCCAGTGCAGGGTCCGAGGTATTCGCACTGGATACGACACAAAG-3′
		FW	5′-CGGCGGTCAGTGCACTACAGA-3′
		REV	5′-GTGCAGGGTCCGAGGT-3′
	5p	Stem–loop	5′-GTCGTATCCAGTGCAGGGTCCGAGGTATTCGCACTGGATACGACAGTCGG-3′
		FW	5′-CGGCGGAAAGTTCTGAGACAC-3′
		REV	5′-GTGCAGGGTCCGAGGT-3′
hsa-miR-148b	3p	Stem–loop	5′-GTCGTATCCAGTGCAGGGTCCGAGGTATTCGCACTGGATACGACACAAAG-3′
		FW	5′-CGGCGGTCAGTGCATCACAGA-3′
		REV	5′-GTGCAGGGTCCGAGGT-3′
	5P	Stem–loop	5′-GTCGTATCCAGTGCAGGGTCCGAGGTATTCGCACTGGATACGACGCCTGA-3′
		FW	5′-GCGCGCGAAGTTCTGTTATAC-3′
		REV	5′-GTGCAGGGTCCGAGGT-3′
hsa-RNU6		Stem–loop	5′-GTCGTATCCAGTGCAGGGTCCGAGGTATTCGCACTGGATACGACACGATT-3′
		FW	5′-CCTGCGCAAGGATGAC-3′
		REV	5′-GTGCAGGGTCCGAGGT-3′
hNRP1		FW	5′-CCCCAAACCACTGATAACTCG-3′
		REV	5′-AGACACCATACCCAACATTCC-3′
hPECAM1		FW	5′-TCAGAAGGACAAGGCGATTG-3′
		REV	5′-GTTATGTTGACCACGATGCTG-3′
hVEGFR2		FW	5′-CCAGTCAGAGACCCACGTTT-3′
		REV	5′-TCCAGAATCCTCTTCCATGC-3′
hGAPDH		FW	5′-CCACCCATGGCAAATTCC-3′
		REV	5′-TCGCTCCTGGAAGATGGTG-3′

### Cell migration assay

To evaluate EC motility, migration assays were performed. For the Transwell migration assay, HUVECs transfected with either the miR-148a/b mimics or the miR-control were starved in M199 media (HyClone) that contained 1% FBS for 6 h. The HUVECs were seeded at 1 × 10^5^ cells/well in the upper chamber of a 24-well Transwell plate that was coated with 0.1% gelatin. The lower chamber contained 600 µL of M199 medium supplemented with 1% FBS and 40 ng of VEGF, which served as a chemoattractant. After 4 h of incubation, cells that did not migrate were removed with a cotton swab. The motile cells were fixed with methanol and stained with hematoxylin and eosin. The motile cells were then counted in each of 8 microscopic regions of interest under 20× magnification using an optical microscope.

### Western blot analysis

HUVECs were washed with cold 1× phosphate-buffered saline and lysed with RIPA buffer (100 mM Tris-Cl, 5 mM EDTA, 50 mM NaCl, 50 mM β-glycero-phosphate, 50 mM NaF, 0.1 mM Na_3_VO_4_, 0.5% NP-40, 1% Triton X-100, and 0.5% sodium deoxycholate) at 4 °C. Sample protein concentration was quantified using a SMART BCA Protein Assay kit (iNtRON). Next, 25 µg of cell lysates was separated by sodium dodecyl sulfate-polyacrylamide gel electrophoresis and then transferred to nitrocellulose membranes. The membranes were blocked with 3% bovine serum albumin in 0.1% TBST, followed by probing with primary antibodies. The membranes were then incubated with horseradish peroxidase-conjugated goat anti-rabbit IgG or goat anti-mouse IgG (Life Science) secondary antibodies. β-actin was used as a loading control. The following primary antibodies were obtained from Cell Signaling Technology and were used at a 1:1000 dilution: NRP1 (Cat. No. 3725), phospho-VEGFR2 (Cat. No. 2478), VEGFR2 (Cat. No. 3479), phospho-FAK (Cat. No. 2541), FAK (Cat. No. 2542), phospho-p38 (Cat No. 9211), p38 (Cat No. 9212), phospho-ERK (Cat. No. 9106), and ERK (Cat. No. 9102). The other primary antibodies used were ROBO1 (1:1000, R&D Systems, Cat No. MAB7118), ITGα5 (1:1000, Santa Cruz Biotechnology, Cat No. sc-10729), and β-actin (1:2000, Thermo Fisher Scientific; Catalog No. MA5-15739).

### F-actin visualization

HUVECs that were transfected with either the miR-148a/b mimics or the miR-control were starved in M199 media that contained 1% FBS for 4 h at 37 °C. The cells were then replated in 35-mm dishes coated with 10 µg/mL fibronectin (Sigma) and were allowed to equilibrate for 4 h. Cells were then stimulated with 20 ng/mL VEGF (Koma Biotech) for 15 min. The HUVECs were then fixed and incubated with rhodamine-conjugated phalloidin-594 (1:250, Invitrogen) and 4′,6-diamidino-2-phenylindole (DAPI; 1:1000, Duolink). Fluorescence images were captured using a Carl Zeiss confocal microscope (LSM700).

### Luciferase miRNA target reporter assay

The NRP1 (NM_003873) 3′ untranslated region (UTR) fragment (4938-5806, 869 base pairs) was cloned using human genomic DNA, and it was inserted into the *XbaI* restriction site of the pGL3-vector at the 3′UTR location (Promega). The amplification primers were as follows:

5′-GCTCTAGAGAATGCTTCTAGAAACTTCCAGC-3′

and 5′-GCTCTAGATACAGTTCAGTTCTATGTGGTTTTTATG-3′.

Luciferase constructs with mutated seed sequences were synthesized using a DNA amplification service from Bioneer. Twenty-four hours after transfection with either the miR-148a/b mimic or the miR-control, the HUVECs were cotransfected with the luciferase constructs and pRL-CMV (a luciferase control reporter vector). The transfected cells were lysed with passive lysis buffer, and luciferase activity was determined using a dual luciferase assay system (Promega).

### Fibrin gel bead assay

A previously established fibrin gel bead assay^15^ was optimized to study angiogenesis. Briefly, 2500 Cytodex beads (GE healthcare) were incubated with 1 × 10^6^ HUVECs for 4 h at 37 °C and 5% CO_2_. The tube was tapped every 20 min for 4 h. The coated beads were then transferred to a T25 flask containing 5 mL of EGM-2 media, where they equilibrated overnight. The following day, the bead-coated cells were resuspended at a concentration of 200 beads/mL in a 2-mg/mL fibrinogen (Sigma) solution containing 0.15 U/mL aprotinin (Sigma) and 0 or 30 ng/mL of hVEGF. Thrombin that was at a concentration of 0.625 U/mL, and it was added to each well of a 24-well plate, followed by the addition of the fibrinogen/bead solution. The plate was incubated at room temperature for 5 min. Then, it was placed in an incubator at 37 °C and 5% CO_2_ for 15 min to generate a clot. During the incubation, human skin fibroblasts were trypsinized and seeded on top of the fibrin gel at a concentration of 20,000 cells/well in 1 mL of EGM-2 supplemented with 2% FBS. The growth media was changed every other day. Sprouting was observed after 2–3 days.

### Cell proliferation assay

Cell proliferation was determined by MTT assay. Briefly, HUVECs were seeded into a gelatin-coated 24-well plate at 3.2 × 10^4^ cells/well and incubated at 37 °C in 5% EC growth medium (EGM-2, Lonza) overnight. After attachment, the cells were cultured with M199 (HyClone) supplemented with 1% FBS. Next, the cells were stimulated with VEGF (Koma Biotech, 20 ng/mL) and EGF (BioLegend, 20 ng/mL) for 24 h. The cells were then incubated for 4 h at 37 °C with MTT solution (0.1 mg/mL, Sigma) for evaluation of cell proliferation. After the 4-h incubation period, the MTT solution was removed, and a 50% dimethyl sulfoxide/ethanol solution (Sigma-Aldrich) was added (200 μl/well) to solubilize formazan crystals. The absorbance was then detected at a wavelength of 540 nm, and cell proliferation was calculated as a percentage of the control.

### Statistical analysis

GraphPad Prism (version 5.1; GraphPad Software, La Jolla, CA) was used for statistical analyses. Statistical significance was determined using the unpaired Student’s *t* test, and *P* values less than 0.05 were considered statistically significant. All experiments were performed at least three times, and representative results are shown. All data are presented as the mean ± standard error of the mean.

## Results

### The expression levels of miR-148a/b-3p are reduced during UCB-MNC to OEC differentiation

**Fig. 1:**
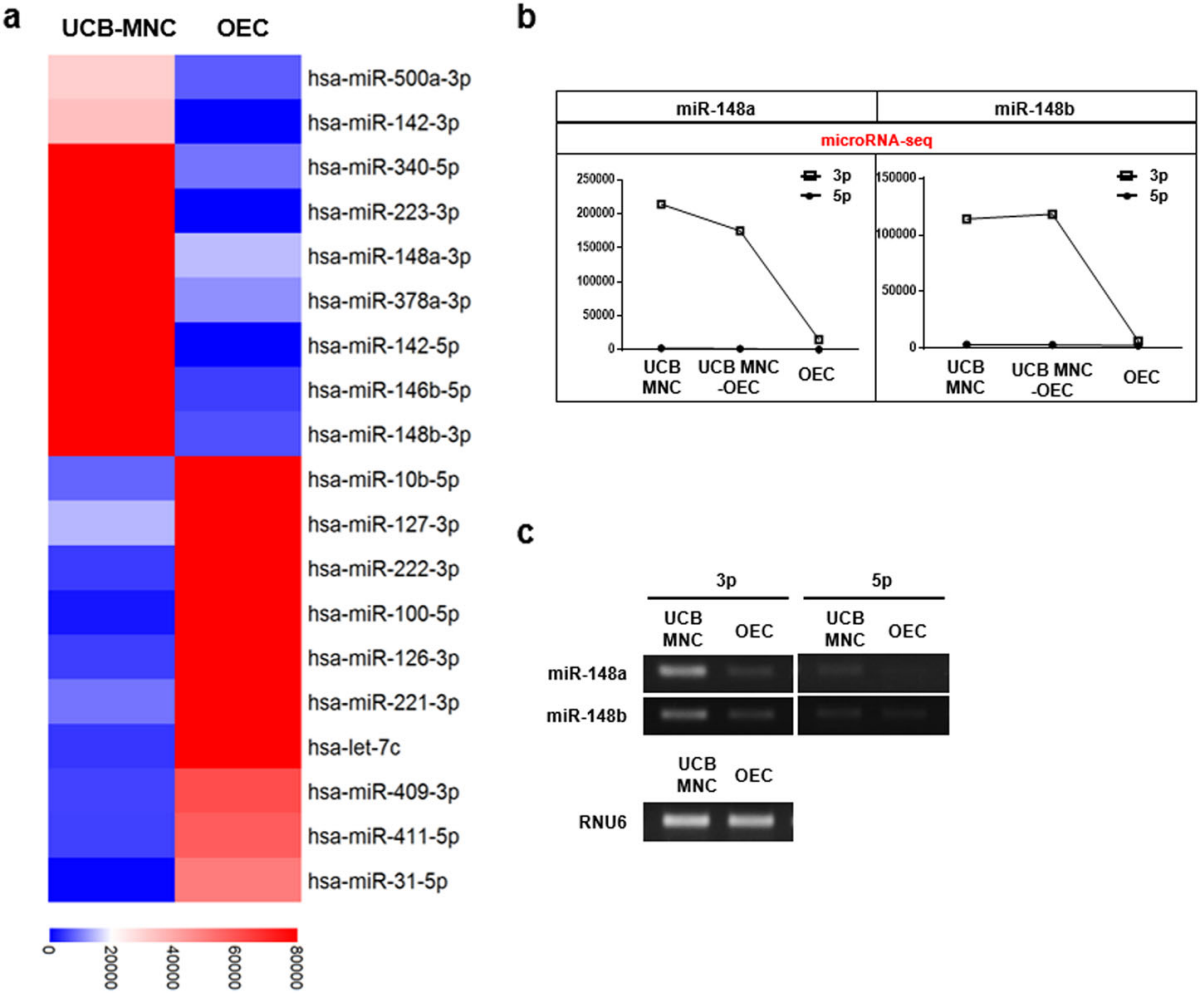
Differentially expressed microRNAs were identified in UCB-MNCs and OECs. **a** A heat map of RNA sequencing data illustrates microRNAs (miRs) that are differentially expressed in UCB-MNCs and OECs. Red and blue indicate high and low-miR expression, respectively. Circulating MNCs were isolated from healthy human umbilical cord blood, and UCB-MNCs were cultured in EBM-2 to induce OEC differentiation. OEC identification and culture purity (90–95%) were determined by the uptake of DiI-conjugated acLDL and by staining with EC-specific markers, including VE–cadherin. **b** Lower levels of miR-148a and miR-148b were observed in OECs than what was observed in UCB-MNCs. **c** The expression of miR-148a/b-3p and miR-148a/b-5p in UCB-MNCs and OECs was confirmed by RT-PCR. RNU6 mRNA was used as an internal control

To identify novel miRs that regulate gene expression in ECs, we conducted microRNA-sequencing (RNA-seq) analysis during UCB-MNC differentiation into OECs. The top 19 differentially expressed miRs were dramatically decreased or increased in OECs compared to their levels in UCB-MNCs (Fig. 1a). Among these, we focused on miR-148a/b-3p because it was significantly downregulated in OECs but had not been studied in ECs (Fig. 1b). We confirmed miR-148a/b-3p expression in UCB-MNCs and OECs using RT-PCR. Interestingly, miR-148a/b-5p was not detected in either UCB-MNCs or OECs. miR-148a/b-3p was significantly downregulated in OECs compared with UCB-MNCs (Fig. 1c). These results indicate that miR-148a/b-3p is downregulated during UCB-MNC differentiation and suggest that miR-148a/b-3p may regulate EC function.

### *NRP1*, *ITGɑ5*, and *ROBO1* are identified as target genes of miR-148a/b-3p

**Fig. 2:**
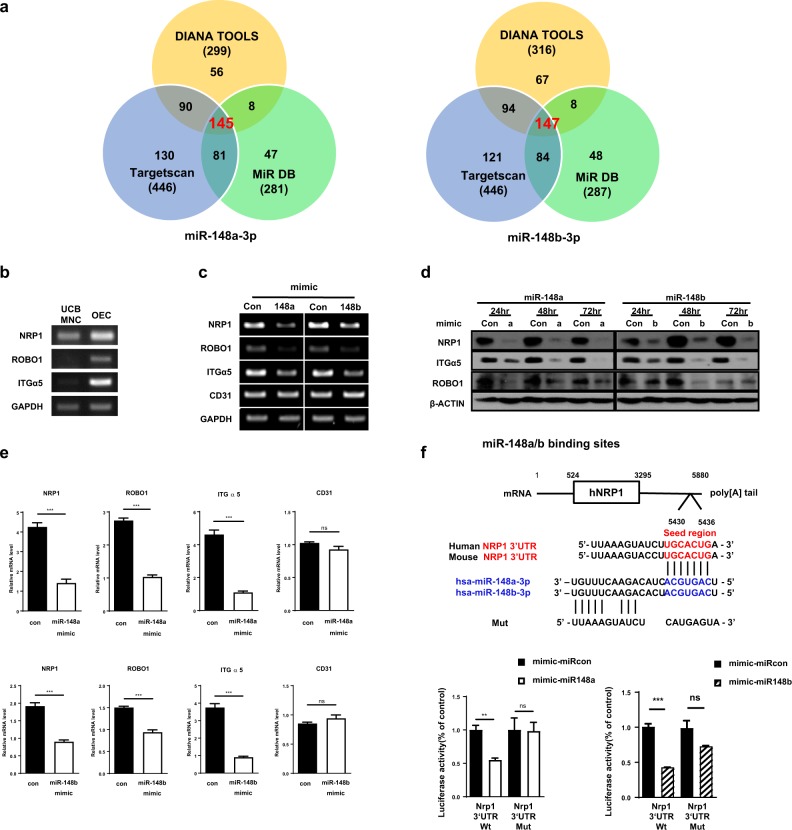
*NRP1*, *ROBO1*, and *ITGα5* were identified as target genes of miR-148a/b-3p. **a** Venn diagrams illustrate the intersection between three algorithms that were used to predict potential gene targets of miR-148a/b-3p. **b** RT-PCR analysis indicates that the expression of *NRP1*, *ROBO1*, and *ITGα5* increased during UCB-MNC to OEC differentiation. **c** The mRNA levels of *NRP1*, *ROBO1*, and *ITGɑ5* in ECs were regulated by miR-148a/b. ECs were transfected with miR mimics (80 µM), and 48 h after transfection, endogenous NRP1 expression was detected by RT-PCR. **d** At 24, 48, or 72 h after transfection, Western blotting analysis was performed to investigate the protein levels of target genes. The internal loading control was β-actin. **e** Relative mRNA expression of *NRP1*, *ROBO1*, and *ITGɑ5* was confirmed by RT-PCR. *PECAM1* expression was unchanged following treatment with the miR-148a/b-3p mimic (*n* = 5 per group). **f** Quantification of the luciferase reporter assay data comparing the wildtype (Wt) NRP1-3′UTR and a mutated (Mut) NRP1-3′UTR; the (Mut) contained a 7-nucleotide mutation in the predicted miR-148a/b-3p binding site. The schematic depicts the predicted miR-148a/b-3p binding site (blue) in the NRP1-3′UTR and shows the 7-nucleotide mutation. All data are presented as the mean ± SEM, **P *<* 0.01, ***P *<* 0.001

We wanted to identify the genes that are regulated by miR148a/b-3p. To identify putative mRNA targets of miR-148a/b-3p, we used three bioinformatic algorithms: DIANA Tools, Targetscan, and miRDB. There were 145 target genes for miR-148a-3p and 147 for miR-148b-3p that were predicted by all three algorithms (Fig. 2a). Of these, *NRP1*, *ITGɑ5* (Integrinα5), and *ROBO1* (Roundabout Guidance Receptor 1) contribute to EC function. To validate the putative miR-148a/b-3p targets, we next investigated the mRNA levels of *NRP1*, *ITGɑ5*, and *ROBO1* in UCB-MNCs and OECs. The expression of *NRP1*, *ITGɑ5*, and *ROBO1* was significantly enhanced in OECs and was inversely correlated with miR-148a/b-3p expression (Fig. 2b). In addition, the mRNA and protein levels of *NRP1*, *ITGɑ5*, and *ROBO1* were repressed by the overexpression of a miR-148a/b-3p mimic in ECs (Fig. 2c, d). Consistent with these data, RT-PCR data revealed that the transfection of a miR-148a/b-3p mimic in ECs reduced the mRNA and protein levels of the target genes in a time-dependent manner (Fig. 2e). Among the identified targets, we focused on NRP1, which is a nontyrosine kinase receptor for VEGF.

To investigate whether NRP1 was a direct target of miR-148a/b-3p, we constructed luciferase reporters containing the 3′UTR of NRP1 mRNA and either the wild-type or mutant seed sequence for miR-148a/b-3p. The luciferase reporters were cotransfected with a miR-control or miR-148a/b-3p mimic into ECs. The reporter assay also indicated that miR-148a/b-3p inhibited NRP1 expression by directly binding to the 3′UTR of NRP1 mRNA (Fig. 2f).

### Upregulation of miR-148a/b-3p delays cell migration and inhibits the formation of lamellipodia in ECs

**Fig. 3:**
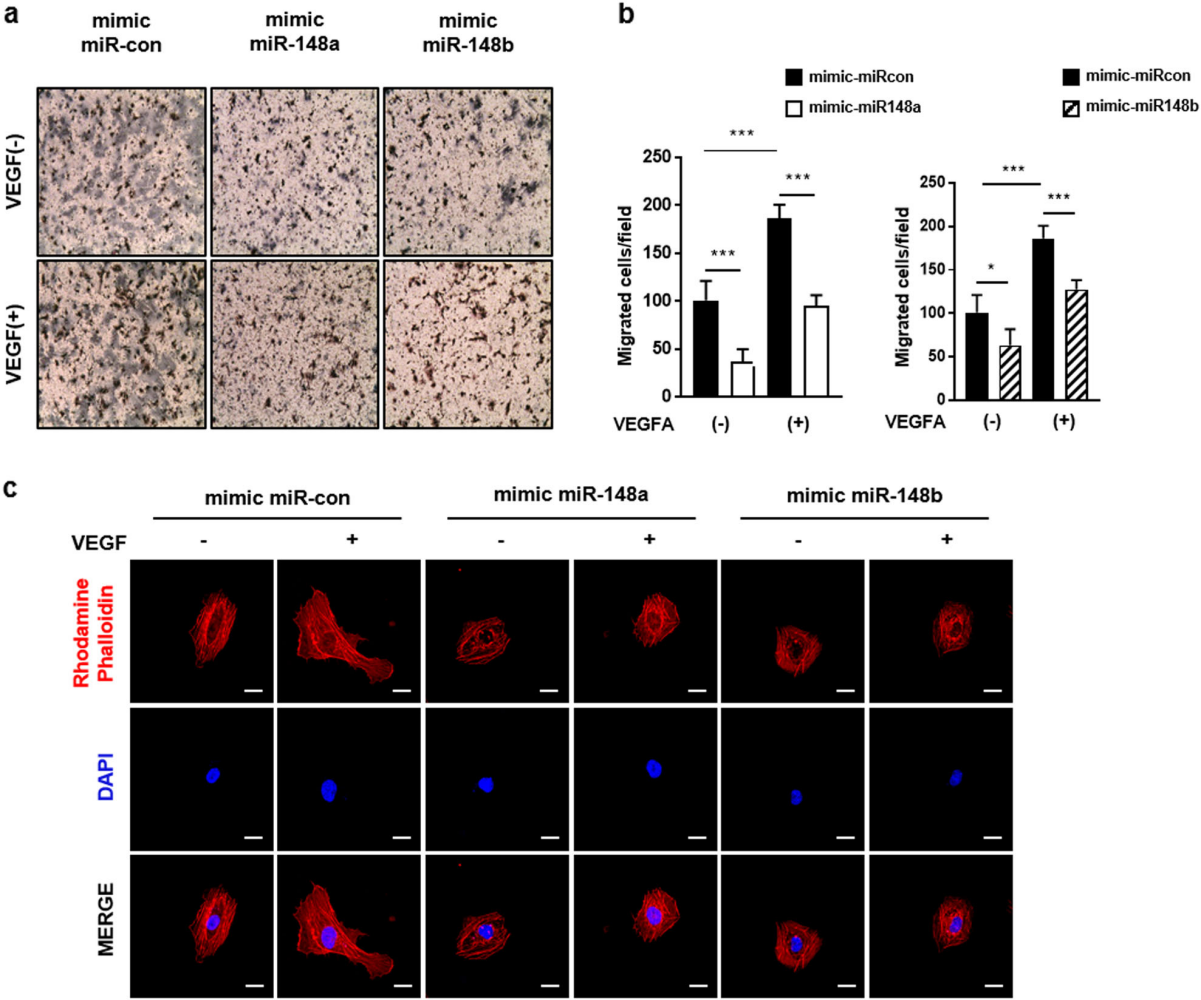
miR-148a/b-3p delayed cell migration and disrupted F-actin remodeling in ECs. **a** Representative microscopic images of ECs that migrated and attached to the bottom membrane during Transwell migration assay. Transfected cells were starved for 6 h in M199 that contained 1% FBS and were then stimulated with VEGF for 4 h. The cells were fixed and stained with hematoxylin and eosin. Images represent cells that migrated within 4 h and were captured; cells were visualized at 20× magnification (*n* = 4 per group). **b** The number of migrated cells per field of view was counted. VEGF-induced EC migration was significantly decreased in ECs treated with the miR-148a/b-3p mimic. **c** Four hour after reseeding on fibronectin-coated dishes, transfected cells were stimulated with 20 ng of VEGF for 15 min. ECs were incubated with rhodamine-conjugated phalloidin to visualize F-actin, and they were incubated with DAPI to visualize nuclei. Images were captured using a Carl Zeiss confocal microscope. Scale bar = 20 µm. Lamellipodia were observed in VEGF-stimulated control cells, but they were not found in miR-148a/b mimic-treated ECs (*n* = 3 per group). All data are presented as the mean ± SEM, *P < 0.05, ^***^P < 0.001

Endogenous expression of miR-148a/b-3p in fully differentiated ECs is low; therefore, we hypothesized that increasing the miR levels in ECs would have a biological effect. We transfected ECs with miR-148-a/b-3p mimics and a miR-control to investigate the effect of miR-148a/b-3p overexpression on angiogenic properties (Supplementary Fig. S1). Because EC migration is an essential step of angiogenesis, we first performed a Transwell migration assay (Fig. 3a). Compared with the control cells, the migration of ECs treated with either miR-148a or miR-148b was reduced by greater than 40%. VEGF stimulation enhanced cell motility, but the migration of ECs treated with the miR-148a/b mimics was less pronounced (Fig. 3b). VEGF-induced EC migration requires remodeling of the actin cytoskeleton into lamellipodia, which prompted us to investigate the effects of miR-148a/b on EC morphology. Phalloidin was used to visualize filamentous actin (F-actin). In control cells, F-actin staining showed lamellipodia with branched actin networks, and tight parallel F-actin bundles were well organized. In addition, after VEGF stimulation, the ends of the actin filaments extended rapidly towards the leading edge (Fig. 3c). These characteristics are typically found in motile cells^16^. On the other hand, the miR-148a/b-3p mimic-treated ECs exhibited a contracted, round morphology, greater cortical actin structures, and fewer F-actin stress fibers. In addition, VEGF stimulation did not induce cellular protrusions toward the leading edge in these mimic-treated cells. We found that miR-148a/b-3p mimic-treated ECs, unlike control cells, failed to migrate through gelatin-coated Transwells, and the cytoskeleton did not remodel into lamellipodia, even with VEGF stimulation. These results suggest that miR-148a/b-3p is an essential mediator of EC migration.

### Overexpression of miR148a/b-3p inhibits angiogenic sprouting in vitro

**Fig. 4:**
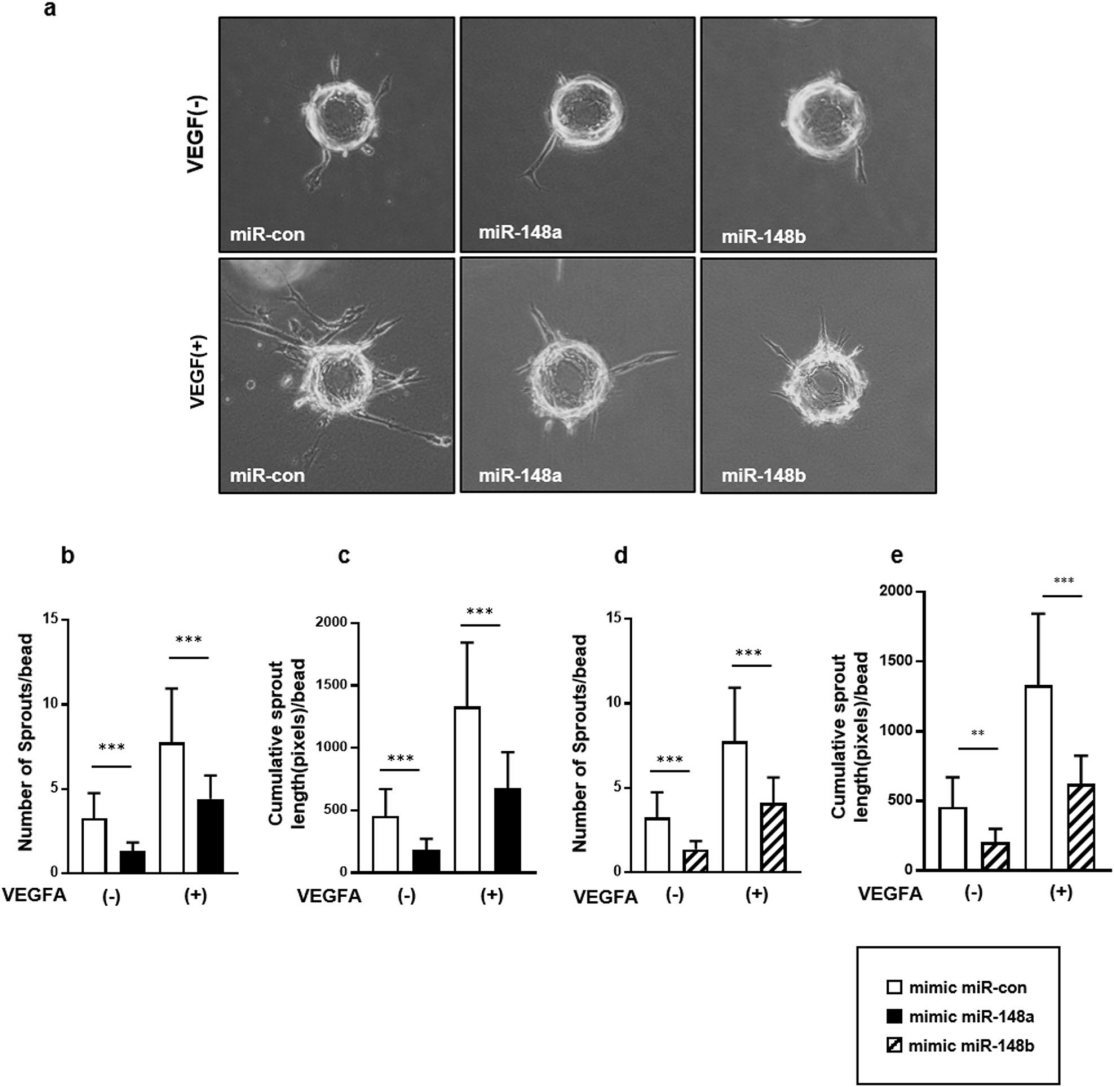
miR-148a/b-3p impaired angiogenic sprouting in fibrin gels. **a** A sprouting assay was performed on HUVECs that were transfected with miR-control or miR-148a/b-3p mimics with or without VEGF (30 ng/mL) stimulation in 2% EBM media. Representative microscopic images show EC morphogenesis during in vitro angiogenesis in fibrin gels. Sprouts were observed on day 2 and continued to proliferate, migrate, branch, and form lumens on days 3–5 in culture. **b** Angiogenic sprouting was quantitatively analyzed by measuring the number of sprouts per bead and the cumulative sprout length per bead (*n* = 10 per group). All data are presented as the mean ± SEM, **P < 0.01, ^***^P < 0.001

Because miR148-a/b-3p inhibited EC migration, we further investigated whether miR148-a/b-3p repressed angiogenesis using a fibrin gel sprouting assay that mimics neovascularization in vitro. miR-148a/b-3p impaired new sprout formation in fibrin gels (Fig. 4a). In the absence of VEGF, ECs transfected with miR148a/b-3p mimics formed fewer sprouts per bead and had a shorter cumulative sprout length when compared to control cells. VEGF stimulation increased the number of sprouts and the cumulative sprout length in both miR-148a/b-3p- and miR-control-transfected ECs (Fig. 4b–e). However, we noted that even with VEGF stimulation, the miR-148a/b-3p mimic-treated ECs had a significantly lower mean number of sprouts and a shorter cumulative sprout length than the control cells did under identical stimulation conditions. These results indicated that miR-148a/b-3p may inhibit the formation of new vasculature.

### VEGFR2 signaling is regulated by miR-148a/b-3p

**Fig. 5:**
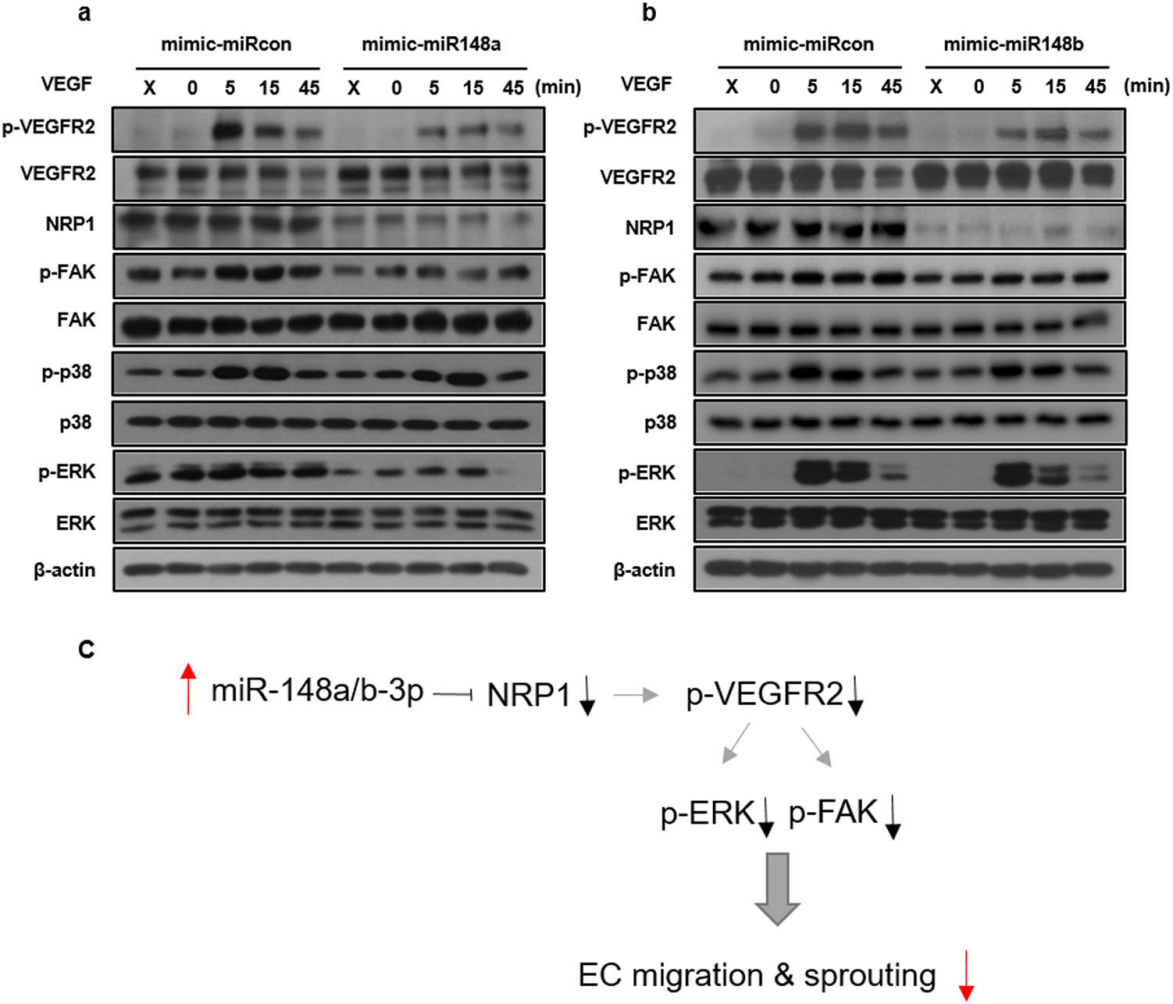
The miR-148a/b-3p mimic inhibited VEGF-induced VEGFR2 kinase activity and downstream signaling in ECs. **a**, **b** Effects of miR-148a/b-3p on VEGFR2 downstream signaling. Western blotting was performed 48 h after ECs were transfected. HUVECs were transfected with the miR-control or miR-148a/b-3p mimics. At 36 h post transfection, cells were starved for 12 h and then stimulated with VEGF (30 ng/mL) for the indicated times. Protein levels were evaluated by Western blot. β-actin was used as an internal control (*n* = 3 per group). Western blot analysis of ECs treated with miR-148a/b-3p mimics indicated a reduction in NRP1 expression. The VEGFR2-mediated activation of FAK, ERK, and p38 was also suppressed by miR-148a/b-3p. **c** Schematic illustration shows the signaling that is regulated by miR-148a/b-3p to inhibit angiogenesis

We next investigated the effects of miR148a/b-3p-mediated NRP1 inhibition on VEGFR2 signaling by stimulating ECs that overexpressed miR-148a/b-3p with VEGF. Although total VEGFR2 protein levels were increased, VEGFR2 and ERK phosphorylation induced by VEGF was decreased (Fig. 5a, b). These data suggest that miR-148a/b-3p exerts antiangiogenic effects by directly targeting NRP1 on the surface of ECs, which disrupts VEGFR2-mediated signaling. Consistent with previous reports, VEGFR2 alone does not fully activate VEGFR2 signaling without its coreceptor, NRP1. Decreased VEGFR2 phosphorylation contributed to the reduced activity of various downstream signaling substrates that are paramount to EC migration and sprouting.

Phosphorylation of other downstream signaling targets of VEGFR2, FAK (focal adhesion kinase) and ERK, was also decreased in cells overexpressing the miR-148a/b-3p mimics (Fig. 5a, b). We observed that the miR-148a/b-3p mimic disrupted lamellipodia formation in ECs (Fig. 3c). Other groups report that in the absence of p-FAK, cells at the edge of a wound do not form protruding lamellipodia. Coupled with our data, these findings suggest that the inhibition of NRP1 expression by miR-148a/b-3p in ECs reduces signaling downstream of VEGFR2 and results in suppressed migration, F-actin remodeling, and sprouting.

## Discussion

ECs are critical to all aspects of vascular homeostasis, and EC dysfunction is directly associated with the development of vascular leakage, infectious diseases, stroke, and cancer angiogenesis^17^. The aim of this study was to investigate the role of miR-148a/b-3p in ECs. miR-148a/b-3p belongs to the miR-148/152 family, which comprises miR-148a, miR-148b, and miR-152^18^. miR-148a and miR-148b, which are 22 nucleotides in length, are conserved in various species, including mice, dogs, and humans (Supplementary Fig. S2). The mature sequence of miR-148a/b has eight nearly identical seed sequences that differ by only two nucleotides. The structural similarity between miR-148a and miR-148b implies that the two miRs may perform similar biological functions. Using RNA-seq analysis, we found that the expression of miR-148a/b-3p significantly decreased during differentiation of UCB-MNCs into OECs. In addition, hsa-miR-142-3p, hsa-miR-223-3p, hsa-miR-146b-5p, hsa-miR-10, hsa-miR-222, and miR-31-5p also showed expression differences that were similar to those observed for miR-148a/b-3p (Fig. 1a). Interestingly, these miRs are known to have angiogenic effects due to their regulation of EC function. For example, miR-142-3p promotes EC proliferation through regulation of Bcl-2-associated transcription factor 1 under hypertensive conditions^19^. miR-223-3p inhibits angiogenesis by suppressing the migration and proliferation of ischemic cardiac microvascular ECs in ischemic heart disease^20^. In addition, upregulation of miR-146b-5p inhibits Ang-1 and causes LPS-induced inflammation in ECs^21^. These prior studies provided foundational evidence that miR-148a/b-3p could have important implications on EC function in angiogenesis. Using miR target prediction programs, we identified 132 common targets for miR-148a/b in both humans and mice. The majority of the gene targets were overlapping and conserved between miR-148a and miR-148b, and they included *NRP1*, *ITGα5*, *ROBO1*, and *DNMT1* (Supplementary Tables 1 and 2). Among these targets, we chose to examine *NRP1* regulation by miR148a/b-3p. The luciferase assay results demonstrated that miR148a/b-3p directly targets the 3′UTR of NRP1. Moreover, we found that the mRNA expression of NRP1 is specifically increased in OECs when compared to what is observed in UCB-MNCs. The expression of miR148a/b-3p was inversely correlated with NRP1 expression. Based on these data, we hypothesized that decreased miR-148a/b expression in OECs would upregulate NRP1 and alter EC function. Thus, we investigated how EC function was changed by increasing miR-148a/b levels. Interestingly, miR148a/b-3p expression caused a decrease in NRP1 expression and led to antiangiogenic phenotypes. We confirmed that the overexpression of miR148a/b-3p inhibited migration and F-actin remodeling in ECs. These findings are consistent with the cell morphology changes that occur when small inhibiting NRP1 RNA (siNRP1) is transfected into human dermal microvascular ECs (HDMECs). In HDMECs, siNRP1 inhibited fibronectin-induced Cdc42 activation and consequently impaired actin remodeling^22^. Furthermore, NRP1 is known to promote EC migration, which is stimulated by both VEGF and extracellular matrix cues^23^. Although it was not a part of the present study, *DNMT1* (DNA [cytosine-5-]methyltransferase 1), was also predicted by our data to be a potential target of miR-148a/b. DNA sequences that encode miRs can undergo abnormal DNA methylation, which influences their expression. In many types of cancers, miR-148a/b expression is low due to the hypermethylation of a CpG island in the miR-148a/b coding region^10,24,25^. In pancreatic cancer, overexpression of miR-148b lowers *DNMT1* expression and contributes to the modified methylation status of tumor suppressor genes^25^. In breast cancer, increased DNMT1 expression lowers DNMT1 expression and contributes to enhanced hyperactivation of the PI3K/AKT and ERK pathways that promote tumor growth^18^. These data support the premise that abnormal expression of miR-148a/b, which regulates DNMT1, promotes tumorigenesis.

Our study revealed that miR-148a/b-3p plays an important role not only in tumorigenesis but also in normal physiological EC function. Indeed, miR148a and miR148b are major regulators of EC migration and sprouting in response to VEGF. As summarized in Fig. 5c, upregulating the expression of miR-148a/b-3p in ECs from its typically low endogenous levels severely inhibited VEGF-induced activation of VEGFR2 and subsequent downstream signaling by directly targeting NRP1. The inhibition of VEGFR2 signaling inhibited migration, F-actin remodeling, and sprouting in ECs. We further confirmed that miR-148a/b-3p markedly reduced EC proliferation through the EGFR signaling pathway, which is also known to regulate NRP1 (Supplementary Fig. S3)^26,27^. Recently, a human IgG1 monoclonal antibody was developed that blocks the NRP1 b1b2 domain, which is in the VEGF binding site, and the antibody was used to treat vascular ECs. Notably, the antibody blocked angiogenesis and vascular remodeling^28^. Collectively, these data suggest that miR148a/b-3p may be regulating antiangiogenic miRs in vascular disease and VEGF-driven angiogenesis by inhibiting NRP1.

## Supplementary information

supplemental material file
