# Supplementary material for: MicroRNA-148a/b-3p regulates angiogenesis by targeting neuropilin-1 in endothelial cells
Source: Exp Mol Med. 2019 Nov 13;51(11):134. doi: 10.1038/s12276-019-0344-x (PMC6853980; doi:10.1038/s12276-019-0344-x)
Supplement: Supplementary file 1 — supplemental material file [file 12276_2019_344_MOESM1_ESM.docx]

Fig. S1


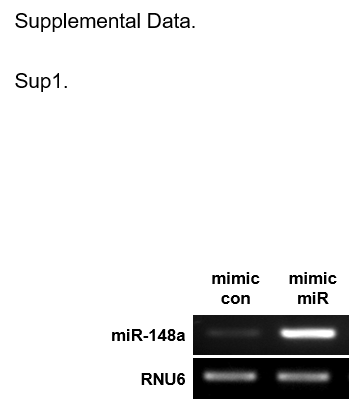


Fig. S2


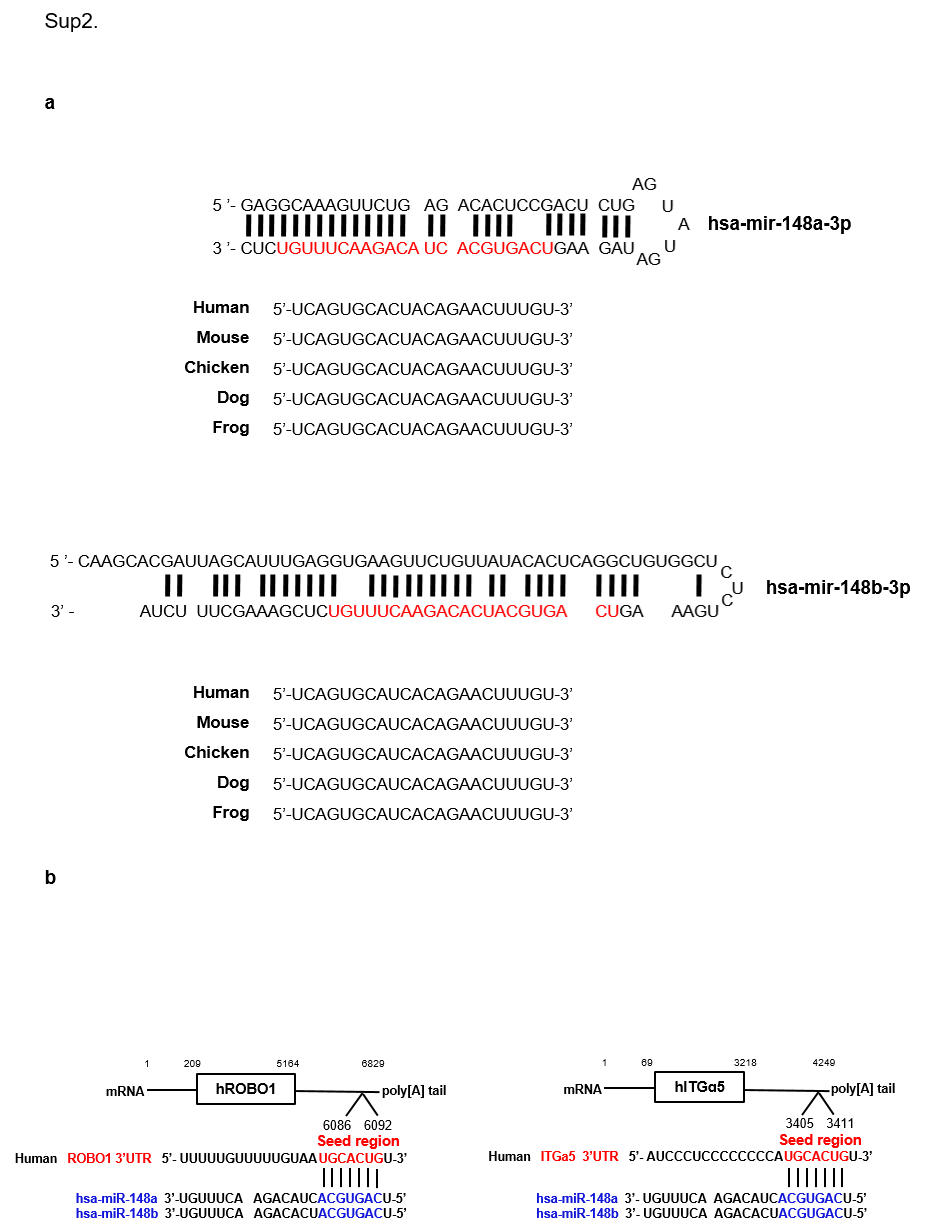


Fig. S3


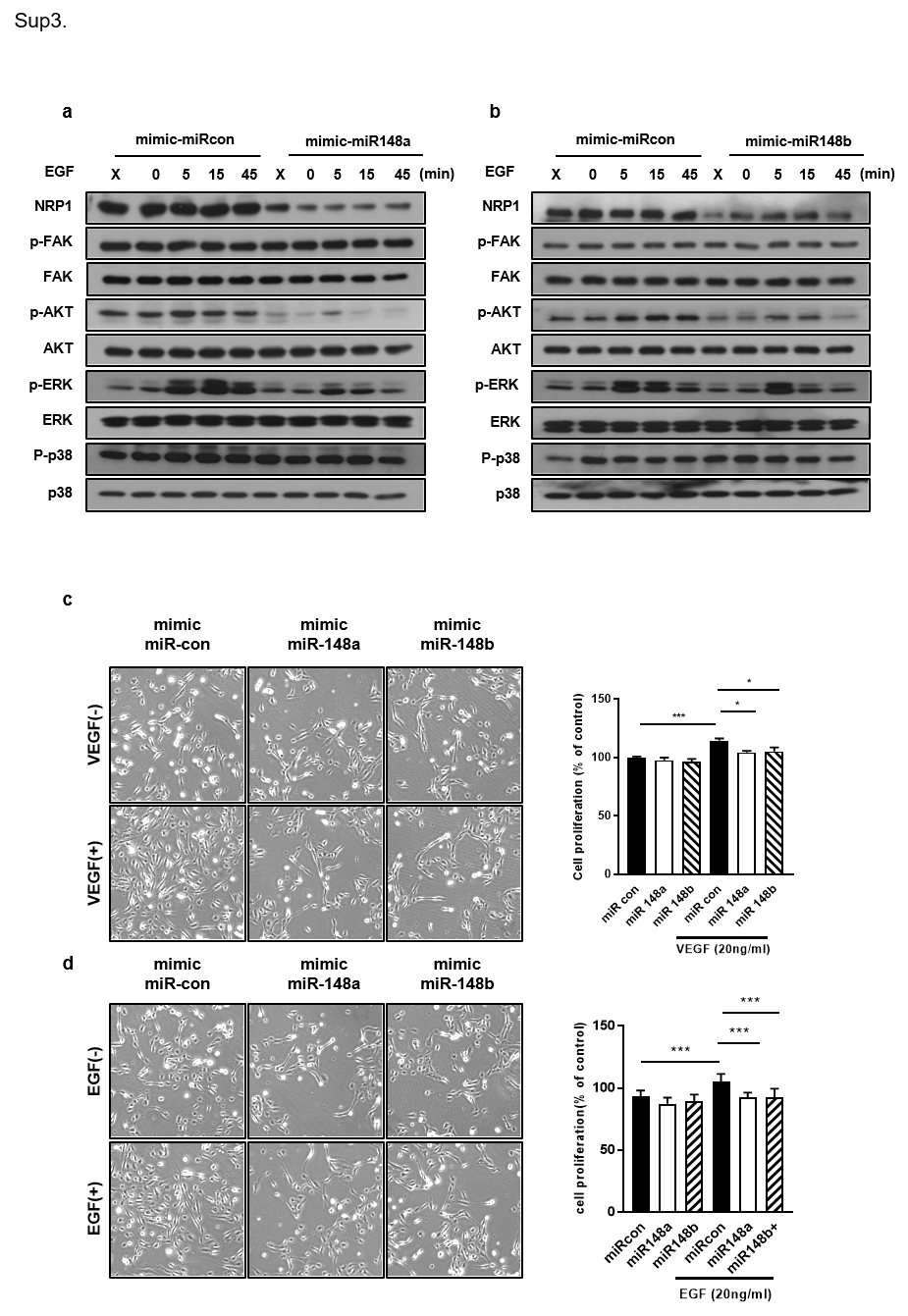


**Supplementary Figure legends**

**Supplementary Figure 1. Overexpression of miR-148a/b by miR-148a/b-3p mimics.**

HUVECs were transfected with miR-control or miR-148a/b-3p mimics (concentration = 80 µM) for 48hrs.

**Supplementary Figure 2. miR-148a/b-3p sequence homology.**

(a) The miR-148a/b-3p sequence is conserved in many species. (b) A putative miR-148a/b-3p binding site exists in the 3’UTR of human *ROBO1* and *ITGα5* mRNA.

**Supplementary Figure 3. miR-148a/b-3p inhibits proliferation of EC induced by VEGF and EGF.**

HUVECs were transfected with miR-control or miR-148a/b-3p mimics. (a-b) Transfected ECs were stimulated with EGF for the indicated times. The miR-148a/b-3p mimic inhibited EGF-induced phosphorylation of AKT and ERK in ECs. (c-d) MTT assay was used to examine the effects of miR-148a/b-3p on cell proliferation. Transfected ECs were treated with VEGF and EGF (20ng/mL) for 24h. All data are presented as the mean ± SEM, **P* < 0.05, *^***^P* < 0.001.

**Supplementary Table 1**. A putative targets list of miR-148a-3p predicted with DIANA-microT-CDS, TargetScan, miRDB

| **miR-148a target prediction** | |
| --- | --- |
| **Abca1** | ATP-binding cassette, sub-family A (ABC1), member 1 |
| **Abcb7** | ATP-binding cassette, sub-family B (MDR/TAP), member 7 |
| **Adam10** | ADAM metallopeptidase domain 10 |
| **Adam22** | ADAM metallopeptidase domain 22 |
| **Adamts15** | ADAM metallopeptidase with thrombospondin type 1 motif, 15 |
| **Agfg1** | ArfGAP with FG repeats 1 |
| **Ago1** | argonaute RISC catalytic component 1 |
| **Ago4** | argonaute RISC catalytic component 4 |
| **Akap1** | A kinase (PRKA) anchor protein 1 |
| **Arfip1** | ADP-ribosylation factor interacting protein 1 |
| **Arhgap21** | Rho GTPase activating protein 21 |
| **Arhgef12** | Rho guanine nucleotide exchange factor (GEF) 12 |
| **Arl6ip1** | ADP-ribosylation factor-like 6 interacting protein 1 |
| **Arl8b** | ADP-ribosylation factor-like 8B |
| **Arrdc3** | arrestin domain containing 3 |
| **Atp11a** | ATPase, class VI, type 11A |
| **Atp2b4** | ATPase, Ca++ transporting, plasma membrane 4 |
| **Atp8a1** | ATPase, aminophospholipid transporter (APLT), class I, type 8A, member 1 |
| **B4galt5** | UDP-Gal:betaGlcNAc beta 1,4- galactosyltransferase, polypeptide 5 |
| **Bach2** | BTB and CNC homology 1, basic leucine zipper transcription factor 2 |
| **Bcl2l11** | BCL2-like 11 (apoptosis facilitator) |
| **Brpf1** | bromodomain and PHD finger containing, 1 |
| **Btaf1** | BTAF1 RNA polymerase II, B-TFIID transcription factor-associated, 170kDa |
| **Btbd3** | BTB (POZ) domain containing 3 |
| **C1galt1** | core 1 synthase, glycoprotein-N-acetylgalactosamine 3-beta-galactosyltransferase, 1 |
| **Cadm1** | cell adhesion molecule 1 |
| **Cand1** | cullin-associated and neddylation-dissociated 1 |
| **Canx** | calnexin |
| **cdk19** | cyclin-dependent kinase 19 |
| **Cdk5r1** | cyclin-dependent kinase 5, regulatory subunit 1 (p35) |
| **Cntn4** | contactin 4 |
| **Col2a1** | collagen, type II, alpha 1 |
| **Cttnbp2nl** | CTTNBP2 N-terminal like |
| **Cul5** | cullin 5 |
| **Cyb5r4** | cytochrome b5 reductase 4 |
| **Cyth3** | cytohesin 3 |
| **Dcp2** | decapping mRNA 2 |
| **Ddx6** | DEAD (Asp-Glu-Ala-Asp) box helicase 6 |
| **Dmxl1** | Dmx-like 1 |
| **Dnmt1** | DNA (cytosine-5-)-methyltransferase 1 |
| **Dock6** | dedicator of cytokinesis 6 |
| **Efnb2** | ephrin-B2 |
| **Elavl2** | ELAV like neuron-specific RNA binding protein 2 |
| **Eogt** | EGF domain-specific O-linked N-acetylglucosamine (GlcNAc) transferase |
| **Epn2** | epsin 2 |
| **Eps15** | epidermal growth factor receptor pathway substrate 15 |
| **Erlin1** | ER lipid raft associated 1 |
| **Fam104a** | family with sequence similarity 104, member A |
| **Fam178a** | family with sequence similarity 178, member A |
| **Fbxo33** | F-box protein 33 |
| **Fmr1** | fragile X mental retardation 1 |
| **Gadd45a** | growth arrest and DNA-damage-inducible, alpha |
| **Gap43** | growth associated protein 43 |
| **Gpm6a** | glycoprotein M6A |
| **Hecw2** | HECT, C2 and WW domain containing E3 ubiquitin protein ligase 2 |
| **Hipk3** | homeodomain interacting protein kinase 3 |
| **Inhbb** | inhibin, beta B |
| **Itga11** | ntegrin, alpha 11 |
| **Itga5** | integrin, alpha 5 (fibronectin receptor, alpha polypeptide |
| **Itsn2** | intersectin 2 |
| **Jarid2** | jumonji, AT rich interactive domain 2 |
| **Jmy** | junction mediating and regulatory protein, p53 cofactor |
| **Jph3** | junctophilin 3 |
| **Kat7** | K(lysine) acetyltransferase 7 |
| **Klf6** | Kruppel-like factor 6 |
| **Kmt2a** | lysine (K)-specific methyltransferase 2A |
| **Lbr** | lamin B receptor |
| **Lipa** | lipase A, lysosomal acid, cholesterol esterase |
| **Lrp2** | low density lipoprotein receptor-related protein 2 |
| **Lrrc41** | leucine rich repeat containing 41 |
| **Ltbp1** | latent transforming growth factor beta binding protein 1 |
| **Maf1** | MAF1 homolog (S. cerevisiae) |
| **Map3k9** | mitogen-activated protein kinase kinase kinase 9 |
| **Med12l** | mediator complex subunit 12-like |
| **Meox2** | mesenchyme homeobox 2 |
| **Mier1** | mesoderm induction early response 1, transcriptional regulator |
| **Mllt10** | myeloid/lymphoid or mixed-lineage leukemia (trithorax homolog, Drosophila); translocated to, 10 |
| **Mmd** | monocyte to macrophage differentiation-associated |
| **Mnt** | MAX network transcriptional repressor |
| **Mras** | muscle RAS oncogene homolog |
| **Mtf1** | metal-regulatory transcription factor 1 |
| **Mtmr12** | myotubularin related protein 12 |
| **Mtmr14** | myotubularin related protein 14 |
| **Naa15** | N(alpha)-acetyltransferase 15, NatA auxiliary subunit |
| **Nog** | noggin |
| **Npepl1** | aminopeptidase-like 1 |
| **Nptn** | neuroplastin |
| **Nptx1** | neuronal pentraxin I |
| **Nrarp** | NOTCH-regulated ankyrin repeat protein |
| **Nras** | neuroblastoma RAS viral (v-ras) oncogene homolog |
| **Nrp1** | neuropilin 1 |
| **Osbpl11** | oxysterol binding protein-like 11 |
| **Otud4** | OTU deubiquitinase 4 |
| **Pdia3** | protein disulfide isomerase family A, member 3 |
| **Phf20** | PHD finger protein 20 |
| **Plaa** | phospholipase A2-activating protein |
| **Pnpla6** | patatin-like phospholipase domain containing 6 |
| **Ppargc1a** | peroxisome proliferator-activated receptor gamma, coactivator 1 alpha |
| **Ppp1cb** | protein phosphatase 1, catalytic subunit, beta isozyme |
| **Ppp1r10** | protein phosphatase 1, regulatory subunit 10 |
| **Ppp1r9a** | protein phosphatase 1, regulatory subunit 9A |
| **Ppp6r1** | protein phosphatase 6, regulatory subunit 1 |
| **Prickle2** | prickle homolog 2 (Drosophila) |
| **Prkaa1** | protein kinase, AMP-activated, alpha 1 catalytic subunit |
| **Prkag2** | protein kinase, AMP-activated, gamma 2 non-catalytic subunit |
| **Pten** | phosphatase and tensin homolog |
| **Ptpn14** | protein tyrosine phosphatase, non-receptor type 14 |
| **Ptprm** | protein tyrosine phosphatase, receptor type, M |
| **Rab34** | RAB34, member RAS oncogene family |
| **Rassf8** | Ras association (RalGDS/AF-6) domain family (N-terminal) member 8 |
| **Rbfox2** | RNA binding protein, fox-1 homolog (C. elegans) 2 |
| **Rbm24** | RNA binding motif protein 24 |
| **Rictor** | RPTOR independent companion of MTOR, complex 2 |
| **Rmnd5a** | required for meiotic nuclear division 5 homolog A (S. cerevisiae) |
| **Rnf38** | ring finger protein 38 |
| **Robo1** | roundabout, axon guidance receptor, homolog 1 (Drosophila) |
| **Robo2** | roundabout, axon guidance receptor, homolog 2 (Drosophila) |
| **S1pr1** | sphingosine-1-phosphate receptor 1 |
| **Sgcb** | sarcoglycan, beta (43kDa dystrophin-associated glycoprotein) |
| **Sgms1** | sphingomyelin synthase 1 |
| **Sik1** | salt-inducible kinase 1 |
| **Slc16a6** | solute carrier family 16, member 6 |
| **Snn** | stannin |
| **Snx27** | sorting nexin family member 27 |
| **Sos1** | son of sevenless homolog 1 (Drosophila) |
| **Sos2** | son of sevenless homolog 2 (Drosophila) |
| **Srsf11** | serine/arginine-rich splicing factor 11 |
| **St8sia3** | ST8 alpha-N-acetyl-neuraminide alpha-2,8-sialyltransferase 3 |
| **Stard13** | StAR-related lipid transfer (START) domain containing 13 |
| **Stk38l** | serine/threonine kinase 38 like |
| **Szrd1** | SUZ RNA binding domain containing 1 |
| **Tbl1xr1** | transducin (beta)-like 1 X-linked receptor 1 |
| **Tgif2** | TGFB-induced factor homeobox 2 |
| **Tmem54** | transmembrane protein 54 |
| **Tmem9b** | TMEM9 domain family, member B |
| **Tnrc6a** | trinucleotide repeat containing 6A |
| **Tnrc6c** | trinucleotide repeat containing 6C |
| **Ube2d1** | ubiquitin-conjugating enzyme E2D 1 |
| **Ucp3** | uncoupling protein 3 (mitochondrial, proton carrier) |
| **Usp48** | ubiquitin specific peptidase 48 |
| **Wasl** | Wiskott-Aldrich syndrome-like |
| **Ythdc2** | YTH domain containing 2 |
| **Ywhab** | tyrosine 3-monooxygenase/tryptophan 5-monooxygenase activation protein, beta |
| **Zdhhc17** | zinc finger, DHHC-type containing 17 |
| **Zfpm2** | zinc finger protein, FOG family member 2 |

**Supplementary Table 2.** A putative targets list of miR-148b-3p predicted with DIANA-microT-CDS, TargetScan, miRDB

| **miR-148b target pridiction** | |
| --- | --- |
| **Abca1** | ATP-binding cassette, sub-family A (ABC1), member 1 |
| **Abcb7** | ATP-binding cassette, sub-family B (MDR/TAP), member 7 |
| **Adam10** | ADAM metallopeptidase domain 10 |
| **Adam22** | ADAM metallopeptidase domain 22 |
| **Adamts15** | ADAM metallopeptidase with thrombospondin type 1 motif, 15 |
| **Agfg1** | ArfGAP with FG repeats 1 |
| **Ago1** | argonaute RISC catalytic component 1 |
| **Ago4** | argonaute RISC catalytic component 4 |
| **Arfip1** | ADP-ribosylation factor interacting protein 1 |
| **Arhgef12** | Rho guanine nucleotide exchange factor (GEF) 12 |
| **Arl6ip1** | ADP-ribosylation factor-like 6 interacting protein 1 |
| **Arrdc3** | arrestin domain containing 3 |
| **Atp11a** | ATPase, class VI, type 11A |
| **Atp2b4** | ATPase, Ca++ transporting, plasma membrane 4 |
| **Atp8a1** | ATPase, aminophospholipid transporter (APLT), class I, type 8A, member 1 |
| **B4galt5** | UDP-Gal:betaGlcNAc beta 1,4- galactosyltransferase, polypeptide 5 |
| **Bach2** | BTB and CNC homology 1, basic leucine zipper transcription factor 2 |
| **Bcl2l11** | BCL2-like 11 (apoptosis facilitator) |
| **Brpf1** | bromodomain and PHD finger containing, 1 |
| **Btaf1** | BTAF1 RNA polymerase II, B-TFIID transcription factor-associated, 170kDa |
| **Btbd3** | BTB (POZ) domain containing 3 |
| **C1galt1** | core 1 synthase, glycoprotein-N-acetylgalactosamine 3-beta-galactosyltransferase, 1 |
| **Cand1** | cullin-associated and neddylation-dissociated 1 |
| **Canx** | calnexin |
| **Cdk19** | cyclin-dependent kinase 19 |
| **Cdk5r1** | cyclin-dependent kinase 5, regulatory subunit 1 (p35) |
| **Chd9** | chromodomain helicase DNA binding protein 9 |
| **Cntn4** | contactin 4 |
| **Col2a1** | collagen, type II, alpha 1 |
| **Cul5** | cullin 5 |
| **Cyb5r4** | cytochrome b5 reductase 4 |
| **Cyth3** | cytohesin 3 |
| **Dcp1a** | decapping mRNA 1A |
| **Dcp2** | decapping mRNA 2 |
| **Ddx6** | DEAD (Asp-Glu-Ala-Asp) box helicase 6 |
| **Dmxl1** | Dmx-like 1 |
| **Dock6** | dedicator of cytokinesis 6 |
| **Efnb2** | ephrin-B2 |
| **Elavl2** | ELAV like neuron-specific RNA binding protein 2 |
| **Eogt** | EGF domain-specific O-linked N-acetylglucosamine (GlcNAc) transferase |
| **Epn2** | epsin 2 |
| **Eps15** | epidermal growth factor receptor pathway substrate 15 |
| **Erlin1** | ER lipid raft associated 1 |
| **Esrrg** | estrogen-related receptor gamma |
| **Fam104a** | family with sequence similarity 104, member A |
| **Fam178a** | family with sequence similarity 178, member A |
| **Fbxo28** | F-box protein 28 |
| **Fbxo33** | F-box protein 33 |
| **Fmr1** | fragile X mental retardation 1 |
| **Foxf1** | forkhead box F1 |
| **Gadd45a** | growth arrest and DNA-damage-inducible, alpha |
| **Gap43** | growth associated protein 43 |
| **Gpm6a** | glycoprotein M6A |
| **Hecw2** | HECT, C2 and WW domain containing E3 ubiquitin protein ligase 2 |
| **Hipk3** | homeodomain interacting protein kinase 3 |
| **Inhbb** | inhibin, beta B |
| **Itga11** | integrin, alpha 11 |
| **Itga5** | integrin, alpha 5 (fibronectin receptor, alpha polypeptide) |
| **Jarid2** | jumonji, AT rich interactive domain 2 |
| **Jmy** | junction mediating and regulatory protein, p53 cofactor |
| **Kat7** | K(lysine) acetyltransferase 7 |
| **Klf4** | Kruppel-like factor 4 (gut) |
| **Klf6** | Kruppel-like factor 6 |
| **Kmt2a** | lysine (K)-specific methyltransferase 2A |
| **Lbr** | lamin B receptor |
| **Lipa** | lipase A, lysosomal acid, cholesterol esterase |
| **Lrp2** | low density lipoprotein receptor-related protein 2 |
| **Lrrc41** | leucine rich repeat containing 41 |
| **Ltbp1** | latent transforming growth factor beta binding protein 1 |
| **Maf1** | MAF1 homolog (S. cerevisiae) |
| **Map3k9** | mitogen-activated protein kinase kinase kinase 9 |
| **Med12l** | mediator complex subunit 12-like |
| **Meox2** | mesenchyme homeobox 2 |
| **Mier1** | mesoderm induction early response 1, transcriptional regulator |
| **Mllt10** | myeloid/lymphoid or mixed-lineage leukemia (trithorax homolog, Drosophila); translocated to, 10 |
| **Mmd** | monocyte to macrophage differentiation-associated |
| **Mnt** | MAX network transcriptional repressor |
| **Mras** | muscle RAS oncogene homolog |
| **Mtf1** | metal-regulatory transcription factor 1 |
| **Mtmr10** | myotubularin related protein 10 |
| **Mtmr12** | myotubularin related protein 12 |
| **Mtmr14** | myotubularin related protein 14 |
| **Mtss1l** | metastasis suppressor 1-like |
| **Naa15** | N(alpha)-acetyltransferase 15, NatA auxiliary subunit |
| **Ncoa1** | nuclear receptor coactivator 1 |
| **Nog** | noggin |
| **Npepl1** | aminopeptidase-like 1 |
| **Nptn** | neuroplastin |
| **Nptx1** | neuronal pentraxin I |
| **Nrarp** | NOTCH-regulated ankyrin repeat protein |
| **Nras** | neuroblastoma RAS viral (v-ras) oncogene homolog |
| **Nrp1** | neuropilin 1 |
| **Osbpl11** | oxysterol binding protein-like 11 |
| **Otud4** | OTU deubiquitinase 4 |
| **Pdia3** | protein disulfide isomerase family A, member 3 |
| **Phf20** | PHD finger protein 20 |
| **Plaa** | phospholipase A2-activating protein |
| **Pnpla6** | patatin-like phospholipase domain containing 6 |
| **Ppargc1a** | peroxisome proliferator-activated receptor gamma, coactivator 1 alpha |
| **Ppp1cb** | protein phosphatase 1, catalytic subunit, beta isozyme |
| **Ppp1r10** | protein phosphatase 1, regulatory subunit 10 |
| **Ppp1r9a** | protein phosphatase 1, regulatory subunit 9A |
| **Ppp6r1** | protein phosphatase 6, regulatory subunit 1 |
| **Prkaa1** | protein kinase, AMP-activated, alpha 1 catalytic subunit |
| **Pten** | phosphatase and tensin homolog |
| **Ptpn14** | protein tyrosine phosphatase, non-receptor type 14 |
| **Ptprm** | protein tyrosine phosphatase, receptor type, M |
| **Rab34** | RAB34, member RAS oncogene family |
| **Rassf8** | Ras association (RalGDS/AF-6) domain family (N-terminal) member 8 |
| **Rbfox2** | RNA binding protein, fox-1 homolog (C. elegans) 2 |
| **Rictor** | RPTOR independent companion of MTOR, complex 2 |
| **Rlim** | ring finger protein, LIM domain interacting |
| **Rmnd5a** | required for meiotic nuclear division 5 homolog A (S. cerevisiae) |
| **Rnf38** | ring finger protein 38 |
| **Robo1** | roundabout, axon guidance receptor, homolog 1 (Drosophila) |
| **Robo2** | roundabout, axon guidance receptor, homolog 2 (Drosophila) |
| **Rsbn1l** | round spermatid basic protein 1-like |
| **S1pr1** | sphingosine-1-phosphate receptor 1 |
| **Sgcb** | sarcoglycan, beta (43kDa dystrophin-associated glycoprotein) |
| **Sgms1** | sphingomyelin synthase 1 |
| **Sik1** | salt-inducible kinase 1 |
| **Slc16a6** | solute carrier family 16, member 6 |
| **Snn** | stannin |
| **Snx27** | sorting nexin family member 27 |
| **Sos1** | son of sevenless homolog 1 (Drosophila) |
| **Sos2** | son of sevenless homolog 2 (Drosophila) |
| **Srsf11** | serine/arginine-rich splicing factor 11 |
| **St8sia3** | ST8 alpha-N-acetyl-neuraminide alpha-2,8-sialyltransferase 3 |
| **Stard13** | StAR-related lipid transfer (START) domain containing 13 |
| **Szrd1** | SUZ RNA binding domain containing 1 |
| **Tbl1xr1** | transducin (beta)-like 1 X-linked receptor 1 |
| **Tgif2** | TGFB-induced factor homeobox 2 |
| **Tmem54** | transmembrane protein 54 |
| **Tmem9b** | TMEM9 domain family, member B |
| **Tnpo1** | transportin 1 |
| **Tnrc6a** | trinucleotide repeat containing 6A |
| **Tnrc6c** | trinucleotide repeat containing 6C |
| **Ube2d1** | ubiquitin-conjugating enzyme E2D 1 |
| **Ube2w** | ubiquitin-conjugating enzyme E2W (putative) |
| **Ucp3** | uncoupling protein 3 (mitochondrial, proton carrier) |
| **Usp48** | ubiquitin specific peptidase 48 |
| **Wasl** | Wiskott-Aldrich syndrome-like |
| **Wnt10b** | wingless-type MMTV integration site family, member 10B |
| **Ythdc2** | YTH domain containing 2 |
| **Ywhab** | tyrosine 3-monooxygenase/tryptophan 5-monooxygenase activation protein, beta |
| **Zbtb18** | zinc finger and BTB domain containing 18 |
| **Zdhhc17** | zinc finger, DHHC-type containing 17 |
